# Study protocol to assess the impact of an integrated nutrition intervention on the growth and development of children under two in rural Bangladesh

**DOI:** 10.1186/s12889-019-7777-y

**Published:** 2019-11-01

**Authors:** Gulshan Ara, Kazi Istiaque Sanin, Mansura Khanam, Shafiqul Alam Sarker, Sihan Sadat Khan, Mahfuza Rifat, Imran Ahmed Chowdhury, Sufia Askari, Kaosar Afsana, Tahmeed Ahmed

**Affiliations:** 1 icddr,b, GPO BOX 128, 68, Shaheed Tajuddin Ahmed Sarani, Dhaka, Bangladesh; 2 BRAC Centre, 75 Mohakhali, Dhaka, 1212 Bangladesh; 3 The Children’s Investment Fund Foundation, 7 Clifford Street, London, W1S 2FT UK

**Keywords:** Nutrition, Intervention, Complementary food, Length-for-age *Z*-score, Stunting, Cognitive development

## Abstract

**Background:**

The period from birth to two years is the “critical window” for achieving optimal growth and development. An inadequate quality and quantities of complementary foods, poor child-feeding practices and infection negatively impact the growth of under-twos. Approximately one-third of under-fives in developing countries are stunted; many are also micronutrient deficient. An estimated 6% of mortalities among under-fives can be prevented by ensuring optimal complementary feeding. The objective of the study was to assess the ability of a 12-month integrated nutrition intervention to improve the nutritional status (length-for-age *Z*-score) of 6 to 12-month-old children in rural Bangladesh.

**Methods:**

In this community-based randomized controlled trial, the intervention group received a package of interventions that includes, food vouchers; to prepare egg-based nutritious snacks (*suji firni* for < 1-year-olds, *suji halwa* for > 1-year-olds), micronutrient powder to fortify children’s food at home, child feeding counselling and water, sanitation and hygiene (WASH), behaviour change communication. The control group received routine health messages provided by the government. Baseline and endline surveys were conducted; Data collection was performed monthly on children’s growth, food voucher utilization, child feeding and morbidity. In addition, we assessed the cognitive development of the children after 12 months of intervention.

**Conclusion:**

This trial aims to explore whether an integrated nutrition intervention can mitigate childhood stunting during the critical window of opportunity in rural Bangladesh. The results may provide robust evidence to improve the linear growth of children in developing countries.

**Trial registration:**

The study was retrospectively registered on August 17, 2018 and is available online at ClinicalTrials.gov (ID: NCT02768181).

## Introduction

Almost half of all deaths among children under five globally can be attributed to undernutrition [1]. Chronic undernutrition results in growth faltering or stunting (i.e., a deficit in length/height relative to age) [2] and is the most prevalent form of undernutrition globally [3]. Approximately 165 million children in low and middle-income countries are estimated to be stunted [1]. Stunting is a leading cause of the global burden of childhood diseases; 80% of which is endured by children in developing countries [4].

The prevalence of stunting peaks during the first 24 months of life. Stunting is a multifactorial problem [5]; however, the higher prevalence of stunting around 2 years-of-age suggests that optimum nutrition and related practices play a pivotal role in development during that age. Hence, this period serves as a critical window of opportunity during which implementation of optimal interventions could reduce the rate of stunting [6].

Complementary feeding is the additional food required by a child, in addition to breast milk, from around the age of 6 months to meet energy and nutrient requirements. Due to limited gastric capacity and higher body demand for nutrition, even children who receive optimal breastfeeding are at risk of stunting if they do not receive sufficient quantities and qualities of complementary feeding after six months-of-age [2]. Approximately 6% of mortalities—equating to 600,000 deaths per year—among under-fives can be prevented by ensuring optimal complementary feeding alone [7]. A recent multi-country analysis involving 21 low-income countries reported that inadequate complementary feeding practices are associated with negative growth patterns [8]. Therefore, improving infant and young child feeding (IYCF) practices has been identified as a fundamental intervention to address to the suboptimal nutritional status of children under five in resource-limited countries [9].

A set of indicators have been defined to assess infant and young child feeding (IYCF) practices to address the feeding-related factors that contribute to suboptimal growth in developing countries [10]. Complementary feeding is one of the major components required to promote child growth and development [11, 12]. Child-feeding practices are multidimensional and change rapidly during the first 2 years. Hence, several factors,—including inadequate quantity, micronutrient deficiencies due to low dietary diversity [13, 14], consumption of low levels of animal-derived foods [15–17] as well as high intakes of known anti-nutrients such as phytates and polyphenols from plant-based foods—play major roles in defining the quality of complementary feeding. The timing and quality of the complementary feeding is so crucial that studies have consistently shown the greatest impact on health outcomes are achieved when food supplementation interventions are delivered to children under 2 years-of-age [18, 19].

Bangladesh has made laudable improvements in several health indicators, in recent decades, with both maternal and child health. Nonetheless, 36% of under-fives in Bangladesh are still stunted, 14% are wasted and 33% are underweight. Furthermore, according to the Bangladesh Demographic and Health Survey, only 23% of children aged 6–23 months are fed a minimum acceptable diet [20], defined as being given milk or milk products and foods from the recommended number of food groups and being fed at least the recommended minimum number of times per day [20]. Multiple studies have made clearly indicated that the quality of complementary feeding in the country is far from adequate [21–25] and that children living in poverty are most vulnerable. The price of nutritious food is a decisive factor in this context, as nutrient-dense foods cost significantly than less nutritious staples such as rice in the Bangladesh, even in comparison to other countries in the region [26].

Global evidence indicates that financial incentives can increase food consumption and improve the nutritional status of new-borns and infants. Evaluations indicated the Colombian Conditional Cash Transfer programmes led to an increase in food expenditure (11% in Mexico, 15% in Columbia) and improved nutritional status among the beneficiaries’ children [27]. A systematic review concluded that cash transfer programmes effectively increase the use of preventive services and sometimes improve the health status of beneficiaries in low and middle-income countries [28]. Evaluation of the Char livelihood program conducted in Bangladesh indicated that women who earned money from the program reported spending more on nutrient-rich food such as animal protein and fruit. Recently, a randomized controlled trial conducted by the International Food Policy Research Institute showed cash transfers combined with nutrition behaviour change communication (BCC) had the largest impact compared to any other transformation modalities of nutrition intervention, leading to a decrease in stunting of 7.3 percentage points, which is nearly three times the national average decline [29].

Considering the prevalence of stunting, its associated factors and the local context in terms of nutritional practices, we proposed a community-based randomized controlled trial to test the impact of an integrated intervention on stunting and the dietary diversity of children under two in rural Bangladesh. The intervention included provision of food vouchers, so mothers could prepare egg-based nutritious snacks for children, distribution of micronutrient powder for fortification of children’s food at home, counselling on child feeding practices and water, sanitation and hygiene (WASH), behaviour change communication (BCC). The overall goal of this trial was to develop and evaluate an intervention strategy that would improve appropriate complementary feeding, as well as corresponding practices such as sanitation and hygiene, in the community and subsequently reduce the burden of stunting among children under two in a poor resource setting.

### Study aim and hypothesis

We hypothesized that an integrated intervention package (provision of food vouchers and micronutrient powder, counselling on child feeding and water sanitation and hygiene, BCC) will improve child growth (length/height) and complementary feeding practices in the selected intervention area of rural Bangladesh compared to the control area.

### Primary objectives of the trial

To improve [1] nutritional status (length for age *Z*-score [30] among children aged 6–12 months through provision of food vouchers and micronutrient powder, counselling on child feeding and water sanitation and hygiene BCC over the 12-month intervention period.

### Secondary objectives of the trial

(1) To improve infant and young child feeding practices in terms of core indicators among children under two-years-old.

## Methods

### Study design

**Fig. 1:**
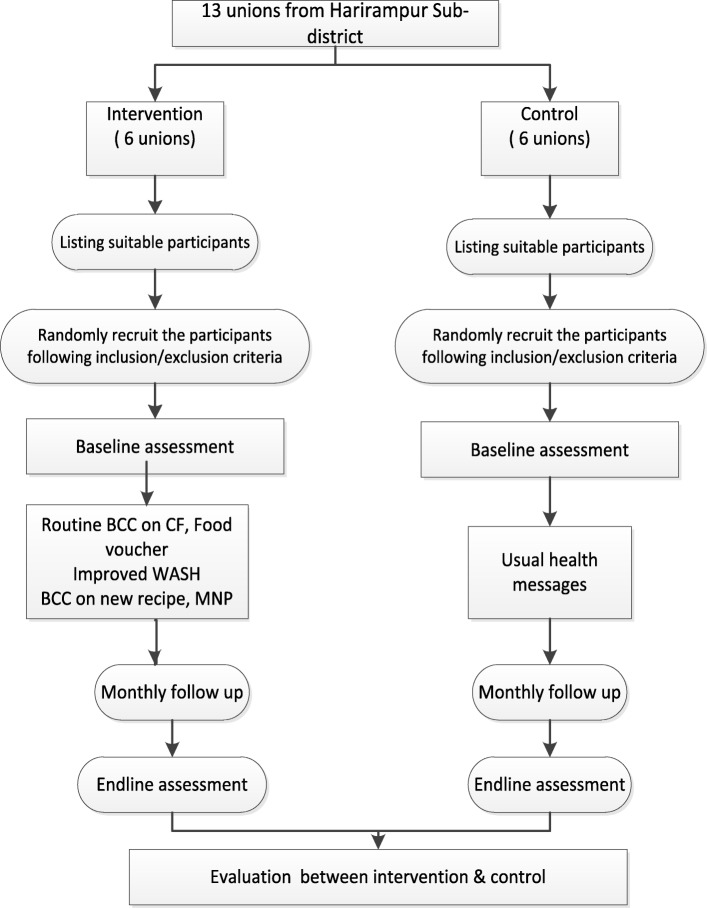
Study flow chart

We planned for a randomized controlled study design with cross-sectional surveys at baseline and endline to examine the effect of our integrated intervention package (provision of food vouchers and micronutrient powder, counselling on child feeding, WASH) to improve child growth and feeding practices. The effect of the intervention package will be compared to a control group in a geographically adjacent area (Fig. 1) with similar population demographics that received the routine health and nutrition related messages provided by the government. The primary outcome assessment will compare the nutritional status of children under-two in the intervention and control groups. Changes in knowledge, attitude and practice regarding child feeding practices among mothers in the intervention and control groups will be assessed and compared between baseline and endline.

### Study site and participants

The study was conducted in the Harirampur sub-district of Manikganj district. The study area was selected purposively in consultation with Bangladesh Rural Advancement Committee (BRAC) in consideration of transportation and logistical issues. Harirampur sub-district contains 13 unions, of which six unions were selected randomly as the intervention area and six unions were selected randomly as the control area (Control Group). Random allocation of the intervention unions was performed by a scientist from the icddr,b who was not involved with this study.

### Sample size and inclusion criteria

The sample size was calculated to identify a difference of 0.4 in the mean LAZ score between the intervention and control groups (− 1.4 vs. -1.8; standard deviation, 1.2) after a 12-month intervention period, with a power of 80%, significance level of 5% and design effect of 1.3. The sample size was calculated as 184 children per arm using a two-tailed *t*-test via the following formula [31]:

$$ \left\{{\left( Z\alpha /2+ Z\beta \right)}^2\times 2{\sigma}^2\right\}/{\delta}^2\times DEFF $$

Where, Z_α/2_ = 1.96, Z_β_ = 0.84, σ = 1.2, δ = 0.4 &DEFF = 1 + λ(*n*-1) where, λ = 0.005 &*n* = 66.

BRAC has a program focusing on households that are too poor to access the benefits of traditional development interventions, which are termed as targeted ultra-poor (TUP) households. This program emphasizes improving the economic and social situation of extremely deprived women and their households who struggle to meet their minimal dietary requirements and face difficulty reaching mainstream anti-poverty programmes such as microfinance. All TUP households (based on BRAC’s other targeted ultra-poor criteria) with children aged 6–12-months-old in the intervention and control areas were identified and listed via door-to-door screening visits. Based on this list which served as our sample frame, 205 mother-child pairs were selected randomly from the intervention unions and enrolled to receive the intervention following the inclusion and exclusion criteria (Table 1). Using a similar approach, 205 mother-child pairs were recruited in the control group and received routine maternal and child health care BCC messages from mass media as per the policy guidelines of the Government of Bangladesh [34].

**Table 1:** Inclusion and exclusion criteria

Inclusion criteria for participants	Exclusion criteria for participants
• Households with children under two (6–12 months during enrolment) • Household listed as a poor household according to the other targeted ultra-poor criteria • Households not involved with any government/non-government microfinance programme • Households not participating in any IYCF programme	Households of children with severe acute malnutrition [32] or a LAZ < − 3 standard deviations of the reference value, as per the WHO guideline [33]

### Criteria for other targeted ultra-poor households used to select participants

- Household depends on a seasonal or irregular income
- Household owns a maximum 30 decimals of land, including homesteads
- Unable to make productive or effective use of NGO or other financial institute’s loans in the past
- Could not afford meat or eggs in any meal in the last two days.

### Intervention delivery platform

BRAC was the implementing institution of this program and employed its extensive network of frontline health workers to deliver the integrated intervention package to the mothers of the children (6–12 months) in the intervention group. A special group of cadres from BRAC known as ***Nutrition Workers*** were solely responsible for delivering the intervention activities. Programme organizers monitored the field activities and supervised the activity of the Nutrition Workers at the field level. The principal investigator (PI), co-investigators (Co-I) and an IYCF counsellor from the icddr,b were responsible for conducting trainings sessions for the Nutrition Workers and routine monitoring and evaluation of the intervention.

### Selection and training of nutrition workers

BRAC recruited total number of eight women from the intervention area as Nutrition Workers; these women had to have at least 8 years of schooling, reside within close proximity to the study area and be motivated to help other mothers to feed their children. Every worker was responsible for delivering the child feeding counselling component of the intervention to 30–35 mothers residing in the same area.

Scheduled home visits were carried out by the Nutrition Workers and one IYCF counsellor. One Nutrition Worker visited each participating mother-child pair at least three times per month to counsel and demonstrate techniques for continuing breastfeeding up to 24 months. Furthermore, special emphasis was given to complementary feeding in terms of the new snack recipes (*suji firni/halwa*), provision of appropriate amounts and frequencies of homemade complementary foods, and appropriate consumption of protein from animal sources based on age for children aged 7–24 months. Mothers were advised to utilize the vouchers provided to purchase the snack recipe ingredients and prepare the snacks at their convenience following the recipe. Nutrition Workers organized demonstrations/cooking sessions with small groups of 10–12 mothers at a time in the intervention groups.

### Snack recipe

A key concern while designing the recipe was to include a source of animal protein in such manner that would be culturally appropriate, locally available and comparatively cheap. An exploratory qualitative study by BRAC indicated semolina, which is locally termed as “suji” is one of the most common food items combined with milk. Thus, investigators from the icddr,b team developed a recipe based on this commonly fed food with addition of an egg, locally known as “*suji firni/suji halwa*. ”*Suji firni* has a semi-solid consistency that is ideal for younger children aged 6–12 months and suji *halwa* was recommended for older children who are able to consume solids (Tables 2 and 3).

**Table 2:** Nutrient content of the egg-based recipe for children aged 6–12 months

*Suji-Firni*	Amount (g)	Energy (kcal)	Protein (g)	Fat (g)	Carbohydrate (g)
Egg	50	69.5	7.25	4.5	–
*Suji*	30	103.8	3.27	0.42	21.18
UHT milk (mL)	200	124	6.55	4.005	5.625
Oil	10	90	–	10	–
Sugar	15	59.7	–	–	14.925
Total		447	17.07	18.925	41.73

**Table 3:** Nutrient content of the egg-based recipe for children aged 13–24 months

*Suji-Halwa*	Amount (g)	Energy kcal	Protein (g)	Fat (g)	Carbohydrate (g)
Egg	50	69.5	7.25	4.5	–
*Suji*	35	121.1	3.815	0.49	24.71
UHT milk (mL)	200	124	6.55	5.34	7.5
Oil	10	90	–	10	–
Sugar	20	79.6	–	–	19.9
Total		484.2	17.615	20.33	52.11

### Food vouchers to obtain ingredients for egg-based recipes for children’s snacks

The sole purpose of the food vouchers was to enable poor mothers to feed their children a nutrient-rich food that provides a source of animal protein and energy, in addition to the child’s regular diet. BRAC provided each mother monthly food vouchers with an equivalent value of BDT 1085 (US$ 13); which were exchanged for the ingredients required to prepare the snack recipes from participating vendors without using cash (Table 4). A number of participating vendors were selected according to pre-determined criteria. The mothers/ caregivers collected suji, oil and sugar once a month and eggs and milk three times a month from the selected vendors. Compliance was monitored by the research staff and programme staff. For programmatic reasons, the purchase was restricted to specific ingredients used to feed children and we expected the voucher system would enable the program to have such control over the purchase.

**Table 4:** Estimated cost of preparing the *suji firni/suji halwa* snack recipes for one child per month

Ingredient	Cost in BDT/month
Milk (6 L, 200 ml/d)	570
Suji (1000 g)	60
Egg (30 @ 11 Tk)	330
Oil (500 mL)	55
Sugar (1000 g)	70
Monthly (BDT)	1085 (US$ 13)

### Behaviour change communication (BCC)

Nutrition Workers used existing, harmonized BCC tools (child-feeding counselling packages including flip charts, videos) to inform mothers about optimum child-feeding practices. However, considering the objectives of the proposed study, a variety of additional BCC materials were developed and incorporated. The core topics of the BCC materials include promotion of the egg-based recipe and water sanitation and hygiene.

Team members from the icddr,b and BRAC organized and conducted hands-on training for all field staff engaged with this project using the national IYCF basic training module. This training focuses on breastfeeding counselling, age appropriate complementary feeding [20], and managing difficulties related to breastfeeding and complementary feeding. Special focus was given to preparation and feeding of the newly developed recipe (*suji firni/halwa)* to the children.

For our study, special child WASH BCC materials were developed to inform and educate mothers on the hazardous effects of ingestion of poultry faces and subsequent environmental enteropathy. No such BCC material has previously been reported in Bangladesh (Table 5). The draft materials were tested in the field to allow mothers and caregivers in the community setting to assess the comprehensibility and clarity of the content. Once the BCC materials were finalized, the icddr,b organized a five-day intensive training for Nutrition Workers on delivering the intervention and motivating the mothers properly. They received training on the newly developed materials, so they could deliver the messages clearly and concisely to encourage mothers to adopt child WASH practices. Additionally, the Nutrition Workers also attended a one-day special refresher training, every two months on cooking process the proposed snacks and feeding practices to address any gaps identified in their routine counselling home visit through monitoring.

**Table 5:** Key messages of the child WASH intervention

Disposal of all faeces, including children’s faeces, in a latrine	
Washing hands with soap after faecal contact and before preparing, eating or serving food	
Keeping children in a clean, protected area where they cannot access dirt/faeces during playtime or mealtimes	
Safe drinking water (collection, transport, storage and treatment)	

### Fortification of children’s meals with micronutrients in the home

Nutrition Workers distributed a one-month supply of micronutrient powder (including iron, vitamin A, vitamin C, folic acid, zinc) during home visits to the mothers in the intervention. They instructed the mothers on how to mix this micronutrient powder with the child’s main meal by dividing the meal into two parts. Mothers were instructed to mix the whole sachet of powder with one half of the meal and feed the child this portion first, then feed the child the remaining portion. Using a counselling card, the Nutrition Workers provided instruction to feed the fortified food within half an hour of mixing to avoid development of a metallic taste and instructed the mothers to use one sachet per meal for one child. Each child was given ten sachets every month, for a year. To ensure compliance, a re-sealable bag was provided to every mother to store the empty powder sachets, which were monitored monthly.

### Intervention duration and outcomes

#### Intervention period

The duration of the intervention was 12 months to ensure there sufficient time to achieve measurable changes in child growth and, IYCF practices.

#### Evaluation plan

A range of quantitative data collection methods including a pre-tested structured questionnaire and anthropometric measurements were adopted to assess the primary and secondary outcomes. A timeline depicting the data collection schedule is given in Table 6.

**Table 6:**
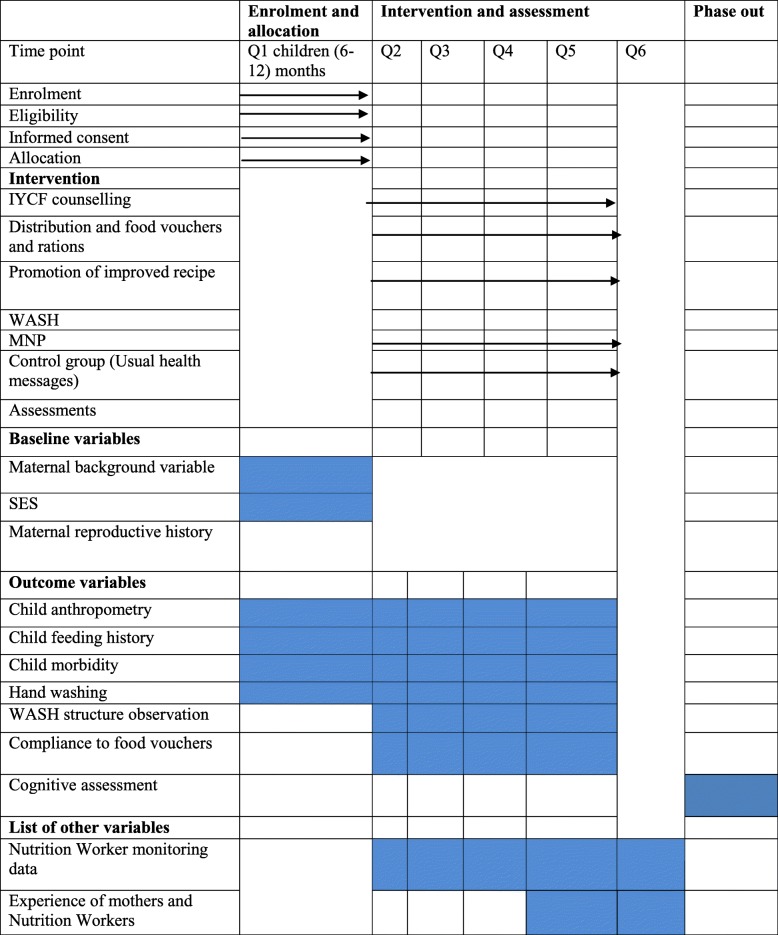
Schedule of study enrolment, interventions and assessments

#### Primary outcome

The primary outcome will be assessed in terms of difference in length between the intervention and control group after a 12-monthintervention period; we anticipate a difference of 0.4 SD in the LAZ score between the intervention and control group.

#### Secondary outcomes

Difference in proportion of children having minimum acceptable diet between the intervention and control group following a 12-month intervention period is our secondary outcome; we anticipate a difference of at least 15%.

#### Measurements

A. *Anthropometry*

Trained field research assistants collected anthropometric measurements (weight and length) monthly following established international methods [35, 36] and recorded these measurements on both the questionnaire, as well as individual child growth charts for the mothers to keep. These research assistants received their training along with the validation exercise at icddr,b. Length of a child was measured to the nearest 1 mm with the help of two staffs using a portable infantometer (Seca 417 model, seca gmbh &co.kg, Hamburg, Germany) [33]. Three measurements were taken consecutively and their average was used. Weight was measured by baby scale (Seca 354 model, seca gmbh & co.kg, Hamburg, Germany) to the nearest 0.01 kg. Similarly 3 measurements were taken and averaged. The measurement tools were checked daily for any possible measurement error before collection of data in the field. We converted the anthropometric measures to z scores for length-for-age (LAZ), weight-for-age (WAZ) and weight-for-length (WLZ) following WHO guideline [37]. Standard WHO recommended indicators were used to assess stunting (LAZ < -2 *Z*), wasting (WLZ < -2 *Z*) and underweight (WAZ < -2 *Z*).

B. *Infant feeding practices*

Standard questions on infant feeding practices previously used in Bangladesh DHS surveys [38] were used to capture IYCF practice during monthly data collection. The questionnaire includes information on current breastfeeding status, current use of other liquids and solid foods, and frequency of consumption. Dietary data were collected using both a 24-dietary recall method and seven-day food frequency questionnaire (FFQ). The standardized data collection tools are capable of assessing core IYCF indicators—such as minimum meal frequency, minimum dietary diversity and minimum acceptable diet [39]. To maintain the quality of data collection, 10% of each interviewer’s scheduled visits were reviewed by a Field Research Officer or the investigators. If the information is incomplete or not clear, the supervisor returns to the same household on the next working day to complete the form.

C. *Infant morbidity*

A history of illnesses such as diarrhoea, dysentery (blood and/or mucus), fever and coughs, ear infections (purulent discharge) from the ears are obtained monthly using a 2-week recall method. These questions are based on the standard DHS infant morbidity recall questions expanded to include questions about ear discharge [20]. Diarrhoea is defined as the passage of three or more loose or watery stools within the last 24 h. The presence of blood in the stools is defined as invasive diarrhoea. A single episode of diarrhoea lasting for more than two weeks is classified as persistent diarrhoea. Acute respiratory illnesses are defined as coughing with reported fast or rapid breathing or difficulty breathing, with or without fever.

D. *Household food security*

Household food security (HFS) information were collected using the Household Food Insecurity access scale (HFIAS) [40].

E. *Hand washing, food safety and structure observation*

A questionnaire is administered solely to assess core WASH indicators. Structured observations are being conducted bi-monthly to assess the regular hand washing practices of mothers and children [41].

F. *Child development*

Mental and psychomotor development data was collected after 12 months intervention using the Ages and Stages Questionnaire (ASQ), Bayley Scales of Infant and Toddler development-III (Bayley-III) and Wolke’s behavior rating scale [42, 43].

#### Process evaluation

A process evaluation focusing on the program’s operation, implementation and service delivery was conducted to assess the fidelity of program implementation. The process evaluation reviews the selection of study participants, implementation of the voucher system, participants’ use of the vouchers, the quality of the different BCC/awareness sessions carried out by the Nutrition Workers, the satisfaction of the staff involved in program implementation, and possible challenges to implementation. Qualitative interviews are planned to be conducted among focus groups of intervention recipients and family members (women, husbands, mothers-in-law) and implementers.

### Data analysis

The primary analyses will compare the mean difference in children’s LAZ scores after the 12-month intervention period using two-sided independent sample *t*-tests at 5% level of error for the group difference. Secondary analyses will examine the core indicators of complementary feeding (minimum meal frequency, minimum dietary diversity, and minimum acceptable diet) in terms of difference in proportions. We will perform intention-to-treat analysis for all inference analyses. We will use generalized liner regression for both continuous and binary outcome with robust Poisson. This will enable analysis of non-normal distribution. All regression models will be adjusted for key covariates known to be associated with the outcome variables. Model assumptions will be checked and appropriate adjustments to the analysis will be made where necessary. We will also perform difference-in-difference analysis to explore the effect of the intervention. STATA® software (STATA version 14.0) will be used for all analyses.

## Discussion

The primary objective of this study was to evaluate the impact of a nutrition-specific community-based randomized-controlled trial intervention on the growth and feeding practices of children under two in rural Bangladesh. According to the Lancet Series on Maternal and Child Undernutrition, implementation of effective targeted nutrition interventions at scale during the window of opportunity (pregnancy and up to 24 months) can reduce the burden of undernutrition-related mortality and disease by 25% [44]. Recommended interventions include promotion of breastfeeding, BCC strategies to improve complementary feeding practices, supplementation and food fortification to improve micronutrient status, health interventions to reduce the burden of infectious diseases among infants and young children, and effective management of severe acute malnutrition. Our trial is the first attempt to incorporate food vouchers to promote adoption of a nutritious egg-based recipe for children’s snacks by mothers in order to improve the quality of complementary food and in turn, child growth. This proposed study design is unique compared to other IYCF interventions, since it covers all necessary components such as BCC, food vouchers for obtaining ingredients, WASH and micronutrient powder for home fortification.

A RCT conducted by Iannoti et al. showed children aged 6–9 months who ate one egg/day for 6 months had significantly higher length-for-age *Z* scores (0.63; 95% confidence interval [CI] [23], 0.38–0.88) and weight-for-age *Z* scores (0.61; 95% CI, 0.45–0.77). Eggs can provide > 50% of the nutrients required by a breastfed child and improve immune function. In addition, the availability of a variety of foods during complementary feeding increases children’s exposure to different varieties of food groups and their acceptance of new foods [45]. BCC interventions have also been shown to improve complementary feeding practices [46]. Through this study, we promoted modified children’s snack recipes (*suji firni/halwa*) via intensive BCC delivered by Nutrition Workers, in an attempt to encourage mothers to prepare and feed their children a culturally acceptable daily snack that contains one egg and 200 ml of milk cooked with semolina, sugar and oil. We anticipated the mothers and caregivers would actively feed children these snacks, and the snacks would not impose an economic burden to the household as all of the ingredients were provided through the food voucher system. We hypothesized that the duration of the intervention (12 months) would be adequate to observe a positive impact on linear growth and cognitive development.

One limitation of this study was our inability to measure the micronutrient status of the children due to funding constraints. Assessment of micronutrient status, especially anaemia and zinc, would allow investigation of the potential contribution of animal protein food sources and micronutrient fortification.

We anticipate the results of this RCT will produce robust evidence on the effectiveness of the egg-based nutritious snacks, BCC and WASH intervention package compared to a control group only be exposed to the usual health messages.

## Data Availability

Data will be publicly available in an accessible format as per icddr,b data policy (https://www.icddrb.org/dmdocuments/icddrb%20Data%20Access%20Policy.pdf).

## Abbreviation

A&T: alive and thrive BCC behaviour change communication BDHS Bangladesh demographic and health survey BARC Bangladesh Rural Advancement Committee CCT Conditional Cash Transfer CF Complementary Feeding CIFF The Children’s Investment Fund Foundation (CIFF) CLP Char Livelihood Project FFQ Food frequency questionnaire GMP Growth Monitoring Promotion HFS Household food security HFIAS Household Food Insecurity access scale IYCF Infant and Young Child Feeding KAP knowledge attitude and practice LAZ length for age *Z*-score PO Programme Organizer RCT Randomized Control Trial TUP Targeted ultra-poor WASH Water, sanitation and hygiene
